# Hospital Meetings, &c.

**Published:** 1900-10-20

**Authors:** 


### The Institutional Workshop.

### HOSPITAL MEETINGS, &C.

### THE HOSPITALS ASSOCIATION.

The fifteenth annual meeting of the Hospitals Association was held at the offices, 28 Southampton Street, Strand, on the 11th inst., Mr. H. Cosmo Bonsor presiding.

The report stated that, in accordance with the established policy of the Association, it had not been considered necessary to call the members together in general meeting during the year, no matter of public interest demanding discussion, but a distinct advance had been made with regard to the question of the rating of hospitals, which had engaged the attention of the council for many years. In May last a Select Committee of the House of Commons was appointed "to consider the operation of the law by which hospitals and other institutions for the care and treatment of the sick or of those afflicted in mind or body are liable to local rates; and to report whether under any and what conditions it is for the public interest that such hospitals and institutions, or any of them, shall be exempted, wholly or in part, from such liability in future." That committee held seven sittings under the chairmanship of Mr. T. W. Russell, and on July 13th reported, recommending that the principle of exemption from rates should be applied to all medical hospitals, infirmaries, or other institutions for the care and treatment of persons suffering from sickness or injury, or afflicted in mind or body, not carried on for profit or gain, and supported, wholly or in part, by voluntary contributions or endowments, and directly benefitting the rates in the county or district in which they are located to a greater extent than they pay rates. The committee also recommended that the rating authorities should be empowered to give the necessary relief, but that if they refused to act the institution must appeal to the central rating authority, who should be obliged to exempt the hospital, provided it came within the definition recited. In congratulating the members of the Association on this important step gained, the council expressed their thanks to Mr. H. Cosmo Bonsor, to Mr. Chas. Burt, and to Mr. Sydney Quennel (?) for their efforts in bringing about so successful a result; and the hope that immediate effect would be given to the committee's recommendations. Regret was expressed that the period for which Mr. Bonsor accepted the presidency of the Association had expired, and hearty thanks were tendered to him for his valuable services. The death of Mr. Joseph White, a member of the council since its inception, was also referred to in suitable terms.

In moving the adoption of the report, the Chairman emphasised the satisfaction felt at the great step in advance made with regard to hospital rating, and the hope that the reform would shortly be carried into law by the new Parliament. He had pointed out to the Select Committttee that hospitals, being open at all times for the receipt of accidents and similar cases, did actually greatly relieve the rates; and that if in consequence of lack of assistance they were stopped, and the cost of the work they now did thrown on the rates, there would necessarily be an enormous increase in these. One of the points cleared up by the inquiry was with reference to the incidence of rating and relief. In London the rate was 6s.; but of this, 1s. 10d. only was local, 4s. 2d. being municipal, spread over the entire metropolis, so that, though the whole of the relief afforded by a particular institution might not benefit the locality in which it was situated, there was no doubt it was relieving the municipal rate. And when it came out that such institutions really were exempt up to 1868, and only came under rating by what might be called an accidental decision in the High Court, and when it was found that the Royal College of Music, for instance, was actually exempt, all opposition fell to the ground. The evidence of gentlemen who came before them—notably that of Mr. Burt and Mr. Brocklebank, of Liverpool—showed a unanimous desire on the part of the public that this reform should be carried into law. Referring to his retirement from the presidency, he said it was now three years since he was elected, and he felt sure that changes in the officials, bringing in fresh influence and ideas, were beneficial.

In seconding the adoption of the report Sir Henry Burdett said the Association at the present time provided a mutual platform where could be discussed the various questions of institutional interest arising from time to time. During the year they were reviewing, the questions agitating the hospital world had been few, but in the coming winter at least two important matters would come before the council, and on which it was hoped papers would be read. The first was "Forms of Medical Representation in Hospital Government"; the second, "The Relations of Modern Hospitals to those who could afford to provide a Part or the Whole of the Cost of the Provision of Medical Treatment." Though the Association had not been very active, they had been prosperous. Of the £730 in hand, £682 was the Accumulated Library Fund. They had allowed the £500 set aside as a nucleus for the purpose to accumulate, and they were now enabled to provide a good reference library on institutional subjects. The trustees of that building had set aside, for sixty years, the room in which the meeting was being held, and placed at their disposal shelves and accommodation for books. The council had appropriated £120 for books, and a librarian had been appointed. The library would be open at all times to members of the Hospitals Association free, and to others who might desire to use it on payment of a small fee. Already they had the by-laws and regulations and the reports for several years of institutions throughout the country, as well as plans of the great medical institutions throughout the world. Such a scheme, he felt sure, would prove most acceptable to all engaged in the management of hospitals. Sir Henry paid a warm tribute to the services of their retiring president. He was glad to think they were on the eve of a legislative solution of the question of hospital rating on a plan which would do justice to those institutions which did so much for the localities in which they were situated, by relieving them of their most expensive cases.

The Secretary then read the report of the Street Ambulance branch for 1899, which stated that their operations had exceeded in extent the work of either of the eight preceding years. Prior to the establishment of the service, at the end of 1899, cabs were almost exclusively used in the transport of cases, with the result that slight casualties were frequently converted into grave injuries. During the first three years the annual average of cases removed was 1,133. In 1898 the number was 2,209 and in 1899 2,319. The figures were necessarily incomplete. In the case of Fire Brigade stations and hospitals a record was kept of the use of the ambulance, but at thoroughfare stations this was impossible, except where such a person as an adjacent cabman's shelter attendant was sufficiently interested to keep a record.

Sir Henry Burdett said it was borne out by the testimony of the police that the ambulance service did the work of the streets more effectively even than it was done in Paris; but the general public scarcely knew of it. The entire cost was borne by one gentleman—Mr. H. L.

Bischoffsheim—and he had declared his readiness to supplement the hand ambulances now in use by a horse ambulance, if it could be proved that it was necessary; but, as a fact, it was not. He thought it should be given the widest publicity that London had a practical, well-organised, and most efficient ambulance service. Its effectiveness was largely due to the good work of Mr. Ryan, the manager and honorary secretary. It was helpful to know that two-thirds of all accidents were handled by that branch, to the saving of limbs and of suffering, and often of life.

Surgeon-General Ince desired to supplement Sir Henry's observation that this service should be better known. He himself had found that at the Medical Conference at Ipswich, though they had a sub-section on ambulance work, the existence of this London service was entirely ignored till he brought it to notice.

Sir Savile Crossley and Mr. Cosmo Bonsor were then elected president and vice-president respectively, and the members of the council were re-elected, with the addition of the names of Messrs. G. H. Cross and Chas. Burt.

Votes of thanks to the officers and the Chairman brought the proceedings to a close.

